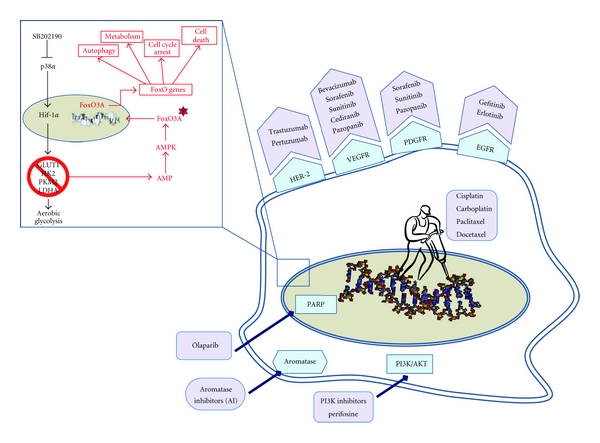# Special Agents Hunting Down Women Silent Killer: The Emerging Role of the p38**α** Kinase

**DOI:** 10.1155/2012/382159

**Published:** 2012-03-11

**Authors:** Valentina Grossi, Cristiano Simone

**Affiliations:** Laboratory of Signal-dependent Transcription, Department of Translational Pharmacology (DTP), Consorzio Mario NegriSud 66030, Santa Maria Imbaro, Italy

## Abstract

Ovarian cancer is sensitive to chemotherapy with platinum compounds; however, the therapy success rate is significantly lowered by a high incidence of recurrence and by the acquisition of drug resistance. These negative outcomes mainly depend on altered apoptotic and drug resistance pathways, determining the need for the design of new therapeutic strategies to improve patient survival. This challenge has become even more critical because it has been recognized that hindering uncontrolled cell growth is not sufficient as the only curative approach. In fact, while current therapies are mostly conceived to impair survival of highly proliferating cells, several lines of research are now focusing on cancer-specific features to specifically target malignant cells with the aim of avoiding drug resistance and reducing adverse effects. Recently, great interest has been generated by the identification of metabolic reprogramming mechanisms occurring in cancer cells, such as the increase in glycolysis levels. In this light, pharmacologic manipulation of relevant pathways involved in cancer-specific metabolism and drug resistance could prove an effective approach to treat ovarian cancer patients.

## 1. Introduction

Ovarian cancer has historically been called the “silent killer,” even if around 80% of patients do actually have symptoms. Indeed, only 20% of ovarian cancers are currently diagnosed while still limited to the ovaries, when up to 90% of patients can be cured using available therapies. Its poor prognosis is related to late diagnosis, which usually occurs at advanced stages, and to acquisition of chemoresistance [1]. To date, more than 30 oncogenes and tumor suppressor genes have been identified that are involved in ovarian oncogenesis inducing modifications in proliferation, apoptosis, anoikis, motility, adhesion, and invasion [2].

## 2. Genetic Alterations in Ovarian Cancer

Although ovarian cancer risk is, at least in part, influenced by hormonal, environmental, and racial factors, a major role is played by genetic factors. Indeed, a key advance in the study of ovarian cancer etiology has been the identification of mutations in the BRCA genes. BRCA1 and BRCA2 genes act as tumor suppressor genes and, when mutated, are associated with the accumulation of chromosomal abnormalities and thus with a higher risk of developing cancer. Inheritance of mutations in BRCA genes is associated with a 27% to 44% lifetime risk of ovarian cancer. A higher incidence of carcinomas of the ovary has also been detected in families affected by the HNPCC syndrome (hereditary nonpolyposis colorectal cancer) [3], which is caused by mutations in DNA mismatch repair genes. HNPCC carriers account for approximately 1% of ovarian cancer patients, and their estimated lifetime risk of ovarian cancer is 9% to 12% [4].

Mutations in BRAF, KRAS, and erbB2 oncogenes and in the tumor suppressor PTEN have been found in a large subset of ovarian cancers [5, 6]. The inactivation of PTEN and an activating mutation of KRAS are sufficient to induce ovarian endometrioid carcinoma in a mouse model [7]. Furthermore, mutations of beta-catenin have been detected both in ovarian carcinomas and in their precursor lesions [8]. Indeed, inactivation of the Wnt/beta-catenin and the PI3K/PTEN pathways has been shown to induce the development of endometrioid carcinoma in an engineered mouse model [9]. The small G-protein RAB25, which regulates motility, aggressiveness, apoptosis, and autophagy and mediates survival in response to stress, has also been found upregulated in the majority of ovarian cancers [10].

The Aurora-A kinase (Aurora-A) is associated with tumor initiation and progression and is overexpressed in various malignancies. Inhibition of Aurora-A induces cell cycle arrest and decreases proliferation of epithelial ovarian cancer stem cells, which represent the chemoresistant population and act as a source of recurrence [11]. All of these and several other amplified oncogenes are potential targets for ovarian cancer therapy.

### 2.1. Chromatin Remodeling and Ovarian Cancer

Molecular genetic changes in chromatin remodeling genes have been identified as a new mechanism in cancer pathogenesis. ARID1A (BAF250a), which promotes the formation of SWI/SNF chromatin remodeling complexes containing BRG1 or BRM, has emerged as a candidate tumor suppressor gene based on its frequent mutations in gynecological cancers. 46%–57% of ovarian clear cell carcinomas, 40% of uterine endometrioid carcinomas, and 30% of ovarian endometrioid carcinomas display somatic sequence mutations in ARID1A [12–14]. Guan and colleagues recently reported that restoring wild-type ARID1A expression in ovarian cancer cells that harbor ARID1A mutations is sufficient to suppress cell proliferation and tumor growth in mice. Moreover, they showed that ARID1A/BRG1 complexes directly interact with p53 and that mutations in the ARID1A and TP53 genes were mutually exclusive in tumor specimens. The regulation of p53-related genes by ARID1A raises the possibility that ARID1A cooperates at the molecular level with p53 to inhibit tumor growth. In non-transformed cells, ARID1A and p53 act as a pair of gatekeepers that prevent tumorigenesis by transcriptional activation of tumor-inhibiting downstream genes, such as CDKN1A and SMAD3. The authors found that all tumors with mutated ARID1A contained wild-type TP53 and tumors with mutated TP53 harbored wild-type ARID1A. Mutations in either ARID1A or TP53 were sufficient to inactivate the ARID1A/BRG1/p53 complex and silence transcription of CDKN1A and SMAD3. This recent study suggests a close collaboration between genetic and epigenetic alterations in cancer pathogenesis [15].

### 2.2. Imprinting and Ovarian Cancer

Genomic imprinting is a molecular mechanism that plays an important role in development, growth, and cell differentiation in mammals. However, only 74 genes have been identified as imprinted among the over 30,000 that can be expressed in human cells. Several of these imprinted genes have been implicated in human oncogenesis. Indeed, while functional inactivation of non-imprinted genes usually requires two genetic alterations, loss of function of imprinted genes may occur following a single genetic or epigenetic event (including loss of heterozygosity (LOH), hypermethylation, and altered transcriptional regulation) occurring on the single functional allele. Moreover, in the case of ovarian oncogenesis, spontaneous mutations may occur during the proliferation of ovarian epithelium to repair ovulatory defects. In this light, downregulation of the imprinted growth-inhibitory genes Aplasia Ras homologue member I (ARHI) and paternally expressed 3 (PEG3) may be particularly important in the pathogenesis of ovarian cancer [16].

ARHI, also known as DIRAS3, is a maternally imprinted tumor suppressor gene encoding a 26 kDa GTPase with 55%–62% homology to Ras and Rap, which inhibits cancer cell growth, motility, and invasion. It is expressed by ovarian epithelial cells and is lost or markedly downregulated in 60%–70% of ovarian cancers [17–19]. Loss of ARHI expression is associated with tumor progression and poor prognosis, while its re-expression in cancer cells inhibits signaling through the Ras/MAPK pathway, induces p21WAF1/CIP1, and downregulates cyclin D1 [19]. Besides, Lu et al. [16] demonstrated that ARHI re-expression causes autophagic death of ovarian cancer cells in culture and participates directly in autophagosome formation by upregulating the ATG4 enzyme that processes the microtubule-associated protein LC3I to LC3II. Autophagy is a process of “self-eating” that involves enzymatic digestion and recycling of cellular constituents in response to stress. While it can contribute to cancer cell death in response to chemotherapeutic agents [20], its role in oncogenesis remains ambiguous as it may also permit survival of cancer cells in response to environmental stress or cytotoxic drugs [21–23]. Indeed, induction of ARHI in xenografts does not kill ovarian cancer cells but instead induces tumor dormancy [24], and its subsequent downregulation rapidly resumes cancer growth.

PEG3 is an imprinted gene encoding a 140 kD Kruppel-type (C2H2) zinc-finger protein that plays an important role in the p53/c-myc-mediated apoptotic pathway. It is significantly downregulated in the majority of ovarian cancers due to promoter hypermethylation and LOH, and its re-expression markedly inhibits ovarian cancer growth. Of note, a high degree of correlation has been found between ARHI and PEG3 in terms of mRNA levels and promoter methylation [25].

## 3. Current Therapies and New Therapeutic Targets

The platinum compounds cisplatin and carboplatin are the most effective chemotherapy agents currently used in ovarian cancer. The antitumor activity of cisplatin (cis-diamminedichloroplatinum (II)) was discovered by Rosenberg and colleagues in 1961 [26]. Cisplatin has been the most active drug used for the treatment of ovarian cancer for the last 4 decades, and response to cisplatin is considered a prognostic factor for patients with ovarian cancer [27]. A high percentage of women with ovarian cancer respond to front-line platinum combination chemotherapy, but in most of them the disease will become resistant to cisplatin, ultimately leading to death [27]. Thus, methods of preventing resistance to cisplatin could prove very useful against ovarian cancer.

The classical therapeutic sequence combines maximal debulking surgery followed by adjuvant platinum- and paclitaxel-based chemotherapy [28, 29]. Unfortunately, 20% of patients do not respond to chemotherapy and recurrent disease occur in >50% of those who initially achieve complete remission, with a 5-year overall survival of only 30%–40% for all stages [30].

New therapeutic approaches based on targeted biologic agents have generated great interest and are currently being investigated in several clinical trials focused on treatments for recurrent ovarian cancer (Figure 1). As is the case for other cancers, angiogenesis is a key process implicated in the metastatization of ovarian cancer. Several growth factors, including vascular endothelial growth factor A (VEGFA), lysophosphatidic acid (LPA), interleukin 6 (IL6), interleukin 8 (IL8), and fibroblast growth factor 1 (FGF1) and 2 (FGF2) are involved in this process [31, 32]. To date, agents that target the VEGF pathway have proven the most effective against the disease.

VEGFA activity has been inhibited by various mechanisms. Bevacizumab, a VEGFA-specific antibody, induced an objective response rate in 16% of patients with recurrent ovarian cancer and stabilized disease for 5.5 months in 50% of patients [33], while improved response rates have been observed in platinum-resistant disease when it was used in combination with cytotoxic chemotherapy [34]. The VEGF Trap is based on a different approach [35]: it is a fusion protein that acts as a soluble VEGF receptor and binds with high affinity to VEGF. Several small molecule inhibitors have been used in ovarian cancer to target VEGF and other pathways. Sorafenib, an oral multikinase inhibitor with activity against Raf and other receptor kinases (including the VEGF receptor (VEGFR), the platelet-derived growth factor receptor (PDGFR), and c-Kit) may have antiangiogenic effects through inhibition of VEGFR. This inhibitor has also been used with promising results in combination with bevacizumab and in combination with chemotherapy, both in recurrent disease and as initial therapy in newly diagnosed patients. Sunitinib is an oral agent that inhibits a number of receptor tyrosine kinases implicated in epithelial ovarian cancer (EOC) growth and metastasis, including VEGFR and PDGFR. It has been assessed in phase II studies for the treatment of advanced or metastatic recurrent EOC [36]. Cediranib (AZD2171) is an oral tyrosine kinase inhibitor with selective activity against VEGFR1, VEGFR2, VEGFR3, and c-Kit. Recent clinical trials showed that cediranib has anticancer activity in recurrent EOC [37]. Pazopanib is an oral angiogenesis inhibitor targeting VEGFR, PDGFR, and c-Kit, which is currently being tested in clinical trials on ovarian cancer.

The epidermal growth factor receptor (EGFR) family is commonly overexpressed in ovarian cancer and has been associated with a negative prognosis; however, limited efficacy has been observed with molecules targeted to the EGFR pathway. Gefitinib and erlotinib, which are inhibitors of EGFR, stabilized disease in 11%–44% of patients with ovarian cancer but produced objective regression in only 4%–6% of cases [38, 39]. The effect of EGFR inhibitors might be reduced by activation of the RAS-MAPK signalling pathway, as happens in colorectal cancers [40]. ErbB2 (also known as HER2) expression in ovarian cancer is associated with advanced stage, higher recurrence frequency, shorter survival time, and lower sensitivity to platinum-based chemotherapy. Trastuzumab and pertuzumab are humanized antibodies targeted against HER2, which act through different mechanisms [41, 42]. In phase II monotherapy clinical studies, trastuzumab has shown activity in certain ovarian cancers overexpressing HER2, while pertuzumab is currently undergoing ovarian cancer trials in combination with cytotoxic agents including gemcitabine [43] and carboplatin [44].

The estrogen receptor *α* (ER*α*) has also been targeted for the treatment of ovarian cancer. Phase II trials of aromatase inhibitors (AIs) have shown modest response but rather better disease stabilization rates, especially when patients are selected on the basis of ER*α* expression [45].

Activation of the PI3K pathway, which occurs in approximately 70% of ovarian cancers, is associated with resistance to cytotoxic chemotherapy. Inhibitors of PI3K and Akt prevent the growth of ovarian cancer xenografts and potentiate the cytotoxic effects of paclitaxel and cisplatin [46]. Perifosine is an alkylphospholipid compound that inhibits Akt and is currently being tested in combination with docetaxel. Development of more specific Akt inhibitors is currently underway and PI3K inhibitors are entering phase I-II trials [47].

Overexpression of IL6 has been detected in the majority of ovarian cancers. It induces a signaling pathway that ultimately stimulates proliferation, inhibits apoptosis, and promotes angiogenesis. Antibodies against IL6 and inhibitors of proteins involved in its pathway, such as JAK2 and STAT3, are currently in development for use in ovarian cancer [48].

Upregulation of the LPA receptors LPAR2 and LPAR3 has been described during the malignant transformation of ovarian surface epithelial cells. An approach targeting this pathway in ovarian cancer cells through antibodies capable of neutralizing LPA and through inhibitors of LPA receptors is currently being studied [49].

Constitutive activation of the NFkB transcription factor has been observed in the majority of ovarian cancers [50, 51]. Activated NFKB induces upregulation of anti-apoptotic genes, growth regulatory cytokines (IL6 or growth regulated *α* (Gro1)), and angiogenic factors (IL8) [52]. A clinical trial is currently underway to study the efficacy of liposomal adenoviral E1A, which interferes with NFKB signaling, in combination with paclitaxel in patients with recurrent ovarian cancer.

The use of poly(ADP-ribose) polymerase (PARP) inhibitors in ovarian cancer is being evaluated in various preclinical, and clinical studies. By interfering with PARP single-strand DNA repair activity, this strategy is aimed at increasing the cytotoxicity associated with DNA damage induced by chemotherapy and takes advantage of the fact that loss of function of BRCA genes, which are also involved in DNA strand breaks repair, is a common feature of this type of cancer [53]. Response to treatment has been observed in 46% of ovarian cancer patients with a BRCA mutation administered with the oral PARP inhibitor AZD2281 (Olaparib) [54]. Moreover, several clinical trials are studying the efficacy of PARP inhibitors in combination with cytotoxic compounds including monofunctional alkylating agents, topoisomerase-I poisons and DNA-crosslinking agents [55].

## 4. p38***α*** and Ovarian Cancer Cell Survival

The high rate of drug resistance acquisition observed in ovarian cancer patients has led to a recent shift in the design of therapeutic strategies: pathways involved in drug resistance are being investigated in depth in order to identify new putative targets, and the potential to manipulate cancer-specific features is being evaluated with the aim of specifically targeting tumor cells in order to reduce adverse effects (Figure 1). As for this second aspect, major attention has been focused on the metabolic reprogramming occurring in cancer cells, which display increased levels of glycolysis compared with their normal counterparts. Indeed, conventional therapies, such as chemotherapy and radiation, produce heavy adverse effects because they are mainly designed to affect survival of highly proliferating cells and thus also damage healthy tissues characterized by a high cellular turnover. In recent years, the observation made in the 1920s by Nobel Prize winner Otto Warburg that tumor cells produce 50% of their adenosine triphosphate through the glycolytic flux versus the 10% observed in normal cells—the so-called Warburg effect—is being revalorized and is now considered a promising target for new therapeutic approaches [56]. This phenomenon is already successfully exploited for the detection of metastasis of most epithelial tumors by positron emission tomography combined with computed tomography (CT; PET/CT) [57, 58]. The Warburg effect seems to be achieved through stable genetic or epigenetic alterations that promote the constitutive activation of the glycolytic pathway and induce a decrease in mitochondrial oxidative phosphorylation, a phenomenon known as aerobic glycolysis. The transcription factor HIF1*α* is one of the central players of cancer-specific aerobic glycolysis. Indeed, its stabilization leads to overexpression of target genes involved in key regulation steps of glucose transport, glycolysis, lactate production, and lactate/proton extrusion [59]. Concomitantly, deregulated HIF1*α* also induces suppression of mitochondrial metabolic pathways, such as oxidative phosphorylation, lipid synthesis, and *β*-oxidation [60]. 

The role of HIF1*α* has been well documented in cancers originating in the ovary. Tumor xenografts obtained from stable HIF1*α*-silenced ovarian cancer cells show increased cell death and necrosis [61], and the expression levels of HIF1*α* have been proposed as an independent prognostic factor in patients with epithelial ovarian tumors [62]. HIF1*α* activity is regulated by several pathways, including the mitogen-activated protein kinase cascade, and p38*α* has been demonstrated to be involved in the stabilization of HIF1*α* in various normal and cancer cell types [63, 64]. The p38*α* pathway regulates proliferation, differentiation, metabolism, and cell death in a cell type-specific and signal-dependent manner [65]. Starting from our promising results obtained on colorectal cancer, showing that p38*α* blockade promotes autophagy, cell cycle arrest, and non-apoptotic programmed cell death both *in vitro* and *in vivo *[66–69], we recently demonstrated that ovarian cancer cells are highly sensitive to p38*α* inhibition [70]. Inhibition of p38*α* activity by the specific inhibitor SB202190 impairs the expression of genes sustaining the altered metabolism of ovarian cancer cell lines and induces a shift from HIF1*α*- to FoxO3A-dependent transcription (Figure 1) [70]. SB202190 promotes a time-dependent reduction of HIF1*α* protein levels, ultimately leading to an acute energy need that triggers the activation of AMPK and the consequent induction of the FoxO3A transcriptional program. In turn, FoxO3A promotes upregulation of crucial mediators of autophagy, cell cycle control, and cell death. Upon p38*α* inhibition, autophagy is first accompanied by G1 arrest, but prolonged inactivation of p38*α* leads to autophagic cell death [70]. Autophagy represents a promising target for the design of new therapeutic strategies relying on pharmacological manipulation in tumors displaying resistance to apoptosis. Besides, as aerobic glycolysis represents a differentiating factor between normal and cancer cells, inhibition of genes involved in cancer cell metabolic reprogramming may provide both specificity and efficacy in countering the energetic demand of transformed cells, thus hampering growth and inducing energy failure-dependent death processes. Thus, therapies based on p38*α*-specific inhibitors could represent a valuable tool against cancer.

The rationale to manipulate the p38 pathway in ovarian cancer is further corroborated by recent findings indicating that p38*α* is a major mediator of drug resistance in response to chemotherapy with 5-fluorouracil and irinotecan [71, 72]. Moreover, as p38*α* inhibitory compounds are currently being investigated in clinical trials for inflammatory diseases and cancer [73], these findings might be taken advantage of in the prospect of clinical translation and support the idea that p38*α* could be one of the special agents engaged by clinicians to hunt down the silent killer.

## Figures and Tables

**Figure 1:**